# Speckle reduction in ultrasound endoscopy using refraction based elevational angular compounding

**DOI:** 10.1038/s41598-021-97717-2

**Published:** 2021-09-15

**Authors:** Parastoo Afshari, Christian Zakian, Jeannine Bachmann, Vasilis Ntziachristos

**Affiliations:** 1 Chair of Biological Imaging, Technical University of Munich, 81675 Munich, Germany; 2 Institute of Biological and Medical Imaging, Helmholtz Zentrum München (GmbH), 85764 Neuherberg, Germany; 3 Department of Surgery, Klinikum Rechts der Isar, Technical University of Munich, 81675 Munich, Germany

**Keywords:** Biomedical engineering, Endoscopy, Ultrasonography

## Abstract

Endoscopic ultrasonography (EUS) is a safe, real-time diagnostic and therapeutic tool. Speckle noise, inherent to ultrasonography, degrades the diagnostic precision of EUS. Elevational angular compounding (EAC) can provide real-time speckle noise reduction; however, EAC has never been applied to EUS because current implementations require costly and bulky arrays and are incompatible with the tight spatial constraints of hollow organs. Here we develop a radial implementation of a refraction-based elevational angular compounding technique (REACT) for EUS and demonstrate for the first time spatial compounding in a radial endoscopy. The proposed implementation was investigated in cylindrical phantoms and demonstrated superior suppression of ultrasound speckle noise and up to a two-fold improvement in signal- and contrast- ratios, compared to standard image processing techniques and averaging. The effect of elevational angular deflection on image fidelity was further investigated in a phantom with lymph node-like structures to determine the optimum elevational angular width for high speckle reduction efficiency while maintaining image fidelity. This study introduces REACT as a potential compact and low-cost solution to impart current radial echo-endoscopes with spatial compounding, which could enable accurate identification and precise sizing of lymph nodes in staging of gastrointestinal tract cancers.

## Introduction

Endoscopic ultrasonography (EUS) is a real-time, minimally invasive diagnostic imaging modality with therapeutic applications in the gastrointestinal (GI) tract region^1–7^, as well as neighboring organs within 4–5 cm of the GI tract, such as the pancreas, liver, and lymph nodes^8–10^. Because of the high spatial resolution and the proximity to the organs, EUS is superior to spiral computed tomography (CT) and magnetic resonance imaging (MRI) for detecting small lesions^11–13^. Thus, EUS is an ideal modality to detect lymph node tumor metastasis, which is crucial for staging of GI tract cancers^8–13^.

Despite these advantages, EUS suffers from poor contrast in the mucosal layers of the GI tract wall^8,9^ due to speckle noise, which is inherent in coherent imaging and arises from the interference of the backscattered waves from tissue microstructures. Speckle noise hinders the identification of tissue-layer boundaries within the GI tract where differences in acoustic impedance are low^10^. Therefore, speckle degrades image quality and contrast, which impedes accurate identification of pathological tissues.

Post-processing techniques to remove speckle from ultrasound images often fail to reveal structures that were obscured by speckle in the original image^14,15^. In contrast, compounding methods can overcome missing information in individual frames by acquiring and averaging a sequence of images containing both correlated features and uncorrelated speckle patterns, with spatial compounding being preferred due to its higher speckle reduction efficiency^14^. To our knowledge, spatial compounding in linear EUS has only been implemented using azimuthal angular compounding^4,10^. Azimuthal angular compounding suffers from limited spatial overlap of images acquired from multiple transmission angles and reduced frame rates due to additional pre-processing for image alignment. Despite its use in linear ultrasound endoscopy, azimuthal compounding is not applicable for radial EUS, as the radial geometry captures the image over a full 360-degree angle in the azimuthal plane, which negates the option of acquiring multiple decorrelated speckle patterns. In contrast, elevational angular compounding (EAC), which relies on capturing partially correlated images by steering the imaging plane with small angular steps in the elevational direction (perpendicular to the imaging plane)^16^, is ideal for radial EUS because its geometry is suitable for capturing sequential frames in a radial configuration. In addition, EAC allows imaging of the same region of interest in all sequential frames, therefore eliminating the need for spatial alignment, which is desirable for real time imaging^16–18^. However, no EAC implementations for EUS have yet been introduced, likely due to spatial and cost constraints. Previous implementations of EAC for traditional ultrasound imaging used either a two dimensional (2D) array^16^ or a mechanical rotating one dimensional (1D) array^17^ to provide the elevational angular imaging; however, 2D arrays are costly and images from mechanically rotating 1D arrays are susceptible to motion artefacts. Moreover, the need for multiple piezoelectric elements in 2D arrays and mechanical stage in rotating 1D arrays make these implementations of EAC bulky and cumbersome, which increases the risk of damage to the GI tract during examination. These factors prevent the translation of EAC to clinical EUS applications.

Our group recently introduced a refraction-based elevational angular compounding technique (REACT), wherein a customized refractive element imparts a fixed linear array with elevational angular steering capabilities^18^. REACT demonstrated more efficient ultrasound despeckling compared to the previous EAC implementations, primarily because the fixed transducer array minimized motion artefacts. However, the refractive element of the REACT prototype was designed for linear arrays, and was therefore not suitable for use in radial ultrasound endoscopy^18^.

In this work, we developed a radial implementation of REACT by using an engraved acoustic cylindrical refractive lens on an annular PMMA substrate to steer ultrasound waves along the elevational angle in cylindrical coordinates. This development represents the first application of spatial compounding in radial EUS. Our radial implementation of REACT achieves elevational angular steering using a stationary 1D-array transducer, making it more compact to avoid potential damage to the GI tract during examination. By integrating radial REACT into a commercially available radial ultrasound endoscope, we image cylindrical layered phantom and demonstrate a two-fold improvement in contrast- and signal-to-noise ratios over uncompounded US images. Moreover, we characterize the optimal elevation angle of deflection for the lymph node- like structures to yield both high speckle reduction efficiency and image fidelity.

## Methods

### Image acquisition

**Figure 1:**
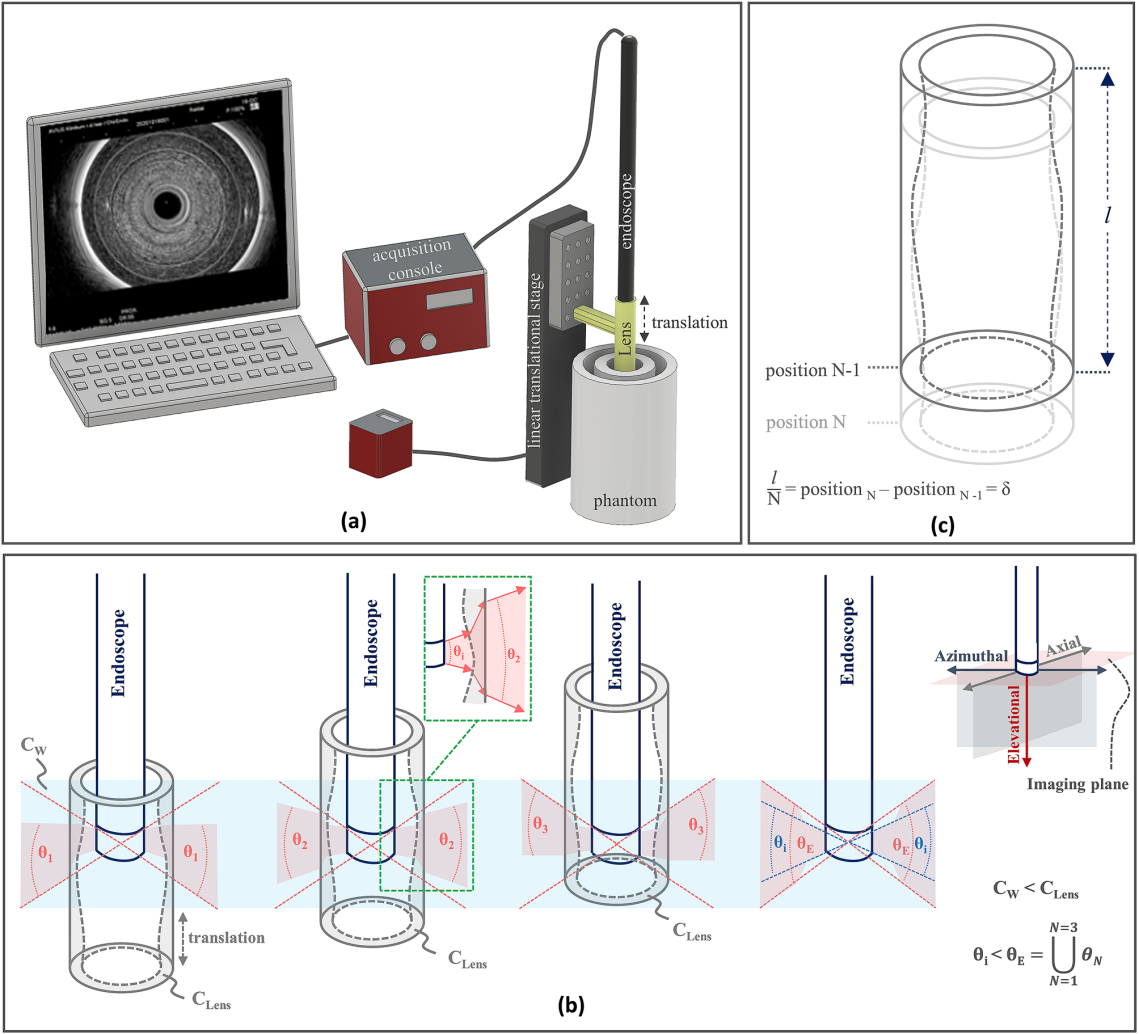
REACT implementation in EUS. **(a)** REACT imaging acquisition configuration using radial transducer array. Linear translation of the annular-shaped acoustic cylindrical lens along the elevational direction in front of the stationary radial transducer array controls the elevational angular FOV. **(b)** Elevational angular steering in different positions of the acoustic cylindrical lens. Sound waves propagate through the water and lens substrate at speeds of c_w_ and c_Lens_, respectively, where c_w_ < c_Lens_. Example of an incidence angle and refracted angle through the lens shown in the inset. The total elevational angular FOV of the transducer (θ_E_: union set of refracted elevational angular views from all positions of the acoustic lens) compared to its inherent elevational angular FOV (θ_i_) is extended. **(c)** Schematic of annular-shaped acoustic cylindrical lens. The annular-shaped acoustic cylindrical lens is moved in consistent step sizes (δ), which are equal to the length of the acoustic lens (*l*) divided by the number of recorded images (N) for compounding.

The implementation scheme of REACT in EUS is shown in Fig. 1a. The employed ultrasound imaging system (HI VISION Avius, Hitachi) utilized a convex radial 360-degree transducer array with central frequency of 7.5 MHz (EUP-R54AW-19, Hitachi). Filters in the software of the EUS system were deactivated prior to capturing images to minimize the amount of pre-processing performed. We acquired all images under the same testing conditions to ensure the validity of the despeckling efficiency comparison between the different refractive lenses. An annular-shaped acoustic cylindrical lens (see below) was attached to a linear translation stage to provide the fixed radial transducer with the elevational angular steering capability. A motorized linear translation stage (MTS50-Z8, Thorlabs) was used to shift the acoustic lens at predetermined linear steps, δ, along the longitudinal axis of the fixed transducer to obtain different elevational angular views by virtue of acoustic refraction (Fig. 1b). Each longitudinal step has an approximate error of 0.7% of the step size. Compounded images were attained by capturing (N = 100) sequential images from the same region of interest, yet at different elevational angular views with a rate of 20 frames per minute. The elevational angular field-of-view (FOV) of the transducer were adjusted by changing the acoustic lens’s position and radius of curvature.

### Refractive element fabrication

Figure 1c shows a schematic of the annular-shaped acoustic cylindrical lens. Customized acoustic cylindrical lenses were manufactured from Polymethyl methacrylate (PMMA), which has a speed of sound of 2750 m/s and a low acoustic attenuation of 1.4 dB/cm/MHz compared to other materials used for acoustic lens fabrication^19,20^. Solid PMMA rods with a diameter of 15 mm ± 4 µm were used as substrate for the acoustic lenses. A 13 mm hole was drilled through the center of the rods to allow a hollow passage for the convex radial 360-degree transducer array. The inner surface of each PMMA tube was later further machined to afford the different curvatures of the refractive acoustic lenses. The acoustic beam refracts in the elevational direction due to the acoustic impedance (Z) difference between the PMMA lens (Z = 3.23 × 10^6^ kg/m^2^s) and water (the imaging medium, Z = 1.49 × 10^6^ kg/m^2^s)^19,20^. The distance between the transducer and cylindrical acoustic lenses was held to ˂1 mm (while avoiding contact) to minimize the effect of multiple reflection artefacts on the image due to the impedance mismatch between the PMMA and water. Depending on the curvature and position in front of the transducer, each cylindrical acoustic lens can provide an extended elevational angle that corresponds to the union set of all angular deflections attained by translating the full length of the lens, *l*, in front of the transducer (Fig. 1c). The specific maximum elevational angular deflection for each lens is achieved when the lens is positioned at the edge of the transducer and all other deflections occur as the lens is translated towards the middle of the transducer. The five manufactured acoustic lenses used in this study provide maximum elevational angular deflections of 0°, 2.5°, 5°, 15°, and 30°. Here, the 0° lens serves as a control case for the refraction-based despeckling principle by yielding the same imaging plane in all relative positions in front of the transducer. To acquire the same number of images for compounding using different lenses, the translation step (δ) for each acoustic lens was selected by dividing the length of each lens (*l*) by the number of acquired images (N = 100; δ: 200 µm, 200 µm, 150 µm, 50 µm, 24 µm, for the 0°, 2.5°, 5°, 15°, and 30° acoustic lenses, respectively). The effective deflection angle was calculated using Snell’s law as c_1_sinØ_2_ = c_2_sinØ_1_ (where c_1_ and c_2_ are the longitudinal wave velocities, and Ø_1_ and Ø_2_ are incidence and exit angles in materials 1 and 2, respectively)^21,22^ and confirmed experimentally in a similar manner to that reported in our previous study^18^. (“See supplementary Fig. 1—Elevational angular characterization of the manufactured lenses using hydrophone and metal target”).

### Imaging samples

Two custom tube-shaped phantoms were manufactured to assess speckle reduction efficiency and image fidelity. They comprised 2% agar and different concentrations of TiO_2_ (0.25–4%) and had outer diameters of 80 mm and inner diameters of 20 mm. Phantom A consisted of five agar layers with TiO_2_ concentrations of 0.25%, 0.5%, 1%, 2%, and 4%, from the outer to the innermost layer, respectively. The phantom contained rod-shaped holes with a 1 mm-diameter to test REACT’s ability to reveal fine structures obscured by speckle noise. Phantom B consisted of a single agar layer with 4% TiO_2_ concentration with embedded spherical water beads with diameters ranging between 10 and 12 mm to mimic lymph nodes-like structures in the GI tract^23^. Phantom B was used to determine the optimum elevational angular width needed for imaging lymph nodes to achieve both high speckle reduction efficiency and image fidelity.

### Analysis method

**Figure 2:**
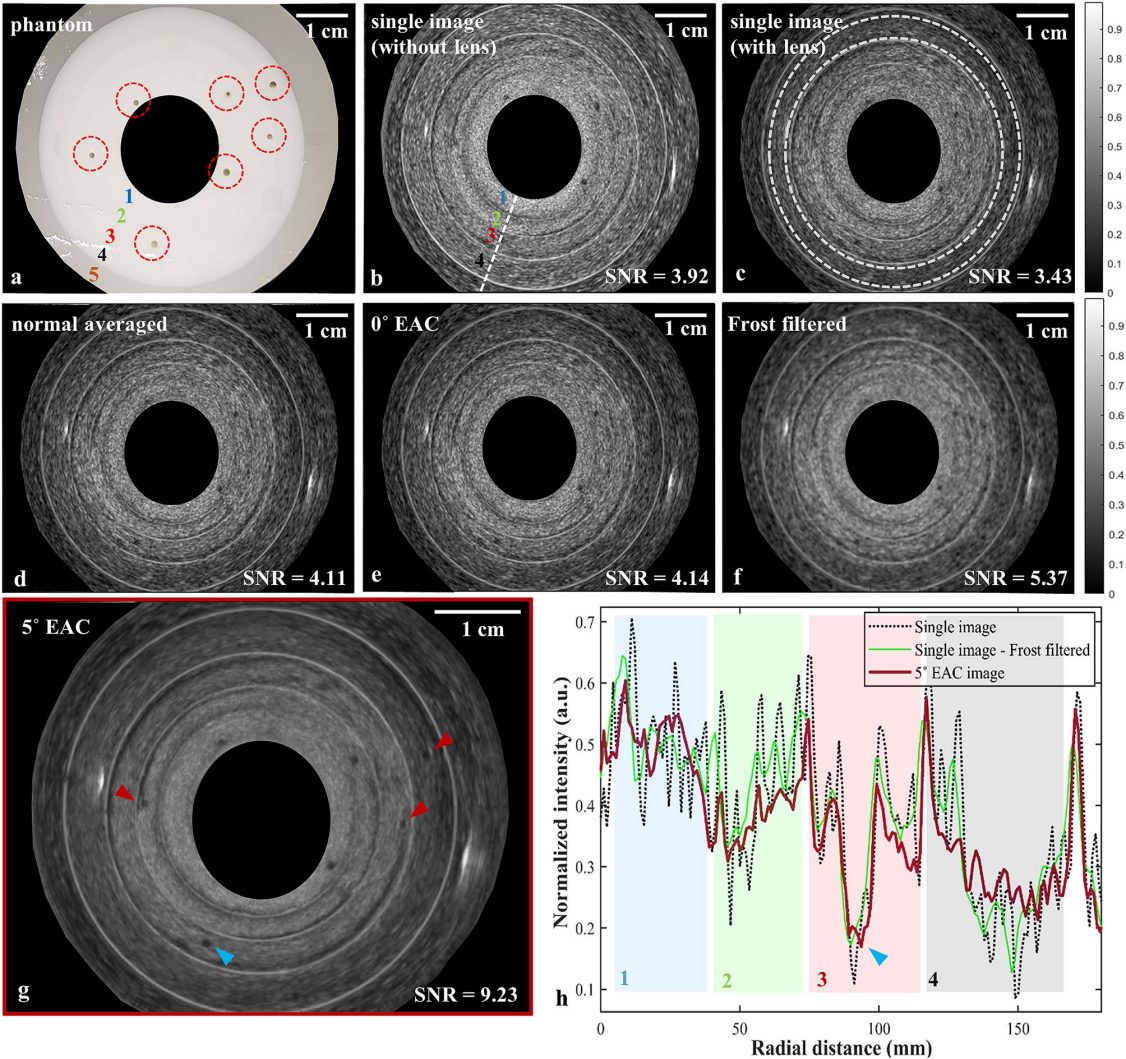
Speckle reduction by REACT in a radial geometry compared to single images, averaging, and Frost filtering. **(a)** Phantom A: a five-layered cylindrical phantom (labelled with numbers 1, 2, 3, 4, and 5 from inside out respectively) containing seven holes (specified with circular regions). **(b–g)** US images of Phantom A: **(b)** a single image recorded without an acoustic lens, **(c)** a single image recorded with a stationary acoustic lens, **(d)** an averaged image using 100 single images recorded with a stationary acoustic cylindrical lens **(e)** a compounded image using 100 single images recorded with a translating acoustic cylindrical lens providing 0° elevational deflection, **(f)** a single image recorded with an acoustic lens after Frost filtering, **(g)** a compounded image using 100 single images recorded with a translating acoustic cylindrical lens providing 5° elevational deflection. The red arrows indicate holes that are not visible in b-f. Region between two white circles in c show the ROI used to derive the SNR in **(b)–(g)**. **(h)** A-line intensity profiles for the single, Frost filtered, and 5° EAC images, which were recorded along the dashed line shown in b. The blue, green, red, and gray rectangles indicate phantom layers 1, 2, 3, and 4, respectively. Blue arrows in g and h point to the same hole, which lies on the dashed line in **(b)**.

**Figure 3:**
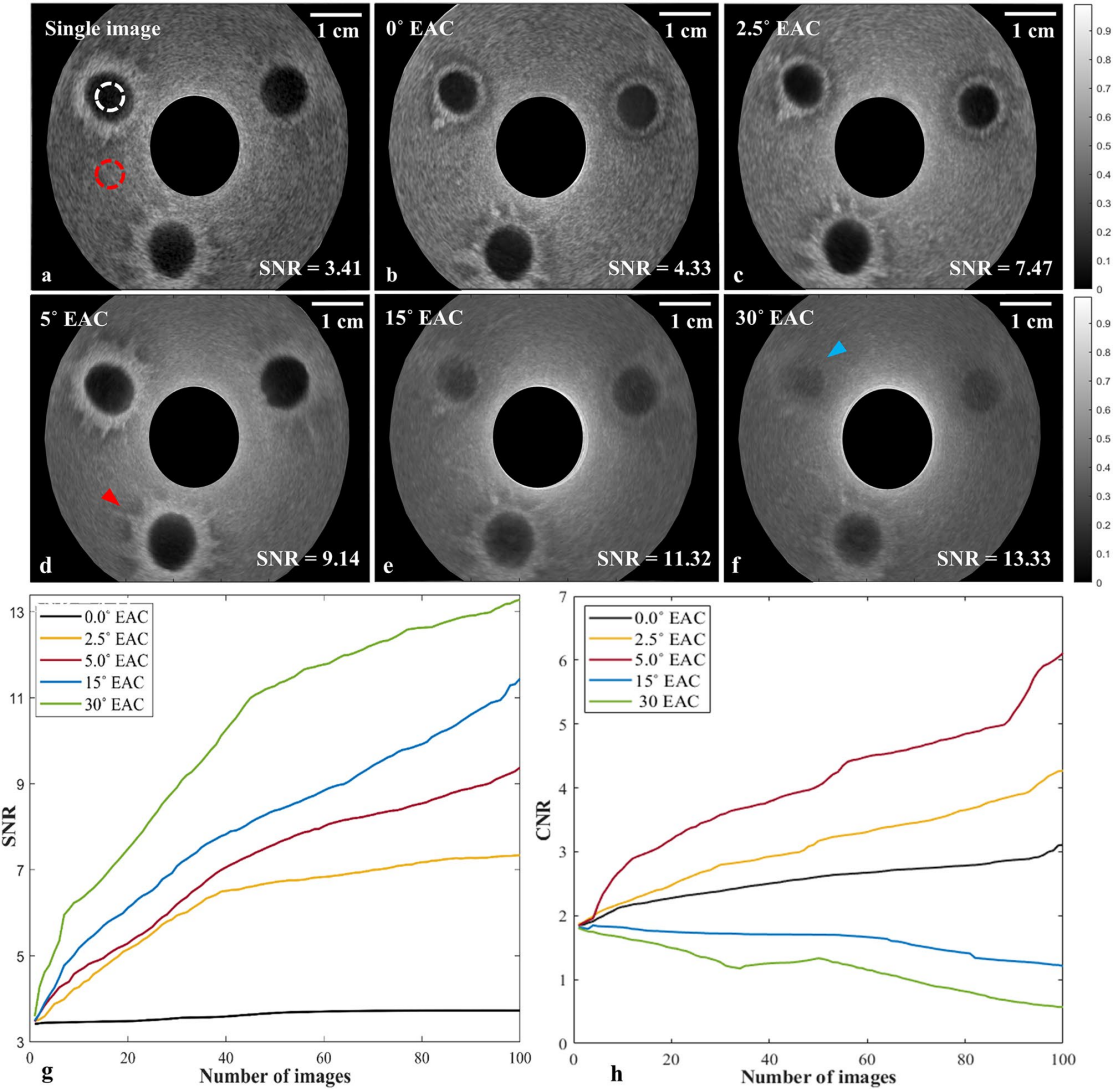
The effect of elevational angular deflection on speckle reduction efficiency and image fidelity in REACT. **(a)** A single US image of a single-layered cylindrical phantom (Phantom B) containing spherical water beads to mimic lymph nodes. The white dotted circles indicate the ROIs used to derive the SNR and CNR. **(b–f)** Compounded images of Phantom B using five different cylindrical acoustic lenses with the following angular deflections: **(b)** 0°, **(c)** 2.5°, **(d)** 5°, **(e)** 15°, **(f)** 30°. The red arrow in d depicts fine structures, which are best resolved by 5° EAC. The blue arrow in f indicates a water bead that is barely visible due to loss of image fidelity at 30° EAC. **(g,h)** The change in SNR and CNR for each deflection angle with increasing number of compounded images.

To attain each compounded image, 100 sequential images were captured in different positions of the annular-shaped acoustic cylindrical lens and compounded using a mean compound operator^24^. To perform image post-processing for despeckling of the US images, MATLAB was used to perform Frost filtering, which is a commonly used speckle noise filtering technique based on an adaptive filter reported in the literature^25–27^. Processed and compounded images were compared to their respect single images to assess the despeckling efficiency. Circular regions of interests (ROI = 13,500 pixels in Fig. 2c, ROIs = 1950 pixels in Fig. 3a) within a water sphere and solid regions in the phantom were selected to derive the average (µ) and standard deviation (σ) of the pixel intensities in order to compute signal-to-noise ratio (SNR : µ_Phantom_/σ_Phantom_) and contrast-to-noise ratio (CNR : |µ_water_ − µ_Phantom_|/σ_water_). These indices were used as quantitative indicators of image improvement to evaluate the despeckling efficiency and preserving the image fidelity. The despeckling efficiency was also evaluated by inspecting the A-line intensity profiles of the single, processed, and compounded images.

## Results

In order to evaluate the speckle reduction efficiency of REACT in a radial geometry and verify its advantage over image post-processing, ultrasound images of a five-layered cylindrical phantom containing seven 1 mm-diameter holes (Phantom A, Fig. 2a) using a radial ultrasound endoscope were recorded and analysed. Figure 2b–g show single images recorded without (Fig. 2b) and with (Fig. 2c) an acoustic lens, an averaged image of 100 single images recorded with a stationary acoustic lens (Fig. 2d), a processed image by Frost filtering (Fig. 2f), and compounded images of 100 single images using a translating acoustic lenses providing 0° (as a control, Fig. 2e) and 5° elevational angular deflections (Fig. 2g). Visual inspection of the ultrasound images in Fig. 2b–g reveals noticeable speckle noise reduction with 5° EAC compared to the single images. Furthermore, the boundaries of the layers and edges of the holes in the phantom are clearer upon 5° EAC compared to averaging and Frost filtering, as highlighted by the red arrows in Fig. 2g.

To quantify the speckle reduction performance, we calculated SNR values (Fig. 2b-g, lower right corner) for each image (See “Methods”, Analysis method subsection). To access a higher number of pixels within the same radius for the SNR calculation, a ring-shaped region within the outer layers of the phantom was selected (enclosed by the dashed white circles in Fig. 2c). The single image recorded with the acoustic lens (Fig. 2c) exhibits a slightly lower SNR than recorded without a lens (Fig. 2b) due to the acoustic attenuation and reflection induced by the cylindrical lens substrate. As expected, images are decorrelated by the REACT method as confirmed by the STD maps (see Supplementary Fig. 2). Compounding of 100 images recorded using both a stationary (Fig. 2d) and translating acoustic lens with a 0° elevational angular deflection (Fig. 2e) improved the SNR by 1.20 times compared to the single image (SNR_single_ = 3.43, SNR_0° EAC, stationary (averaged)_ = 4.11, SNR_0° EAC, translating_ = 4.14). The identical SNR measured for the 0° deflection lens both when stationary and translating reflects its non-refractive nature, which leads to all corresponding images being in the same imaging plane (unchanged speckle pattern). However, due to the refractive capability of the 5° elevational angular deflection lens, the SNR obtained with this lens (Fig. 2g, SNR_5°EAC_ = 9.23) was 2.69 times greater than the SNR of the single image (Fig. 2c) and 1.71 times greater the SNR of the Frost filtered image (Fig. 2f, SNR_Frost filtered_ = 5.37). The speckle pattern decorrelation is related to the elevational angular deflection obtained with the movement of the refractive lens. However, the linear translation of the lens results in non-uniform angular deflection across the field-of-view, as confirmed by the speckle pattern decorrelation obtained with the 5° EAC lens (see Supplementary Fig. 2). An optimal number of compounding images would be obtained by linearly translating the lens to positions that deflect the acoustic beam to equidistant angular steps.

Figure 2h shows the A-line intensity profiles (dashed line in Fig. 2b) for a single image recorded with lens (dotted black line), Frost filtered image (solid green line), and the compounded image using 5° EAC (solid red line). The blue, green, red, and gray rectangles in Fig. 2h indicate layers 1–4 of Phantom A, respectively. Inspecting the A-line intensity profile of the single image shows high intensity variations due to presence of scatterers producing speckle noise. Frost filtering affords a slight dampening of the high intensity variations (solid green line), yet it follows the noise pattern of the main signal since it operates only on the available data in a single image. In contrast, these variations are strongly suppressed by 5° EAC (solid red line), regardless of the noise pattern of the original signal. The higher speckle noise suppression in 5° EAC is due to compounding of different speckle patterns acquired at different elevational angular views. The high speckle reduction afforded by REACT is exemplified by a hole that is only reveal upon 5° EAC (blue arrow in Fig. 2g,h).

Figure 3 depicts single and compounded ultrasound images of a single-layered cylindrical phantom containing spherical water beads to mimic lymph nodes (Phantom B), which illustrate the effect of the elevational angular deflection on speckle reduction efficiency and image fidelity for the radial implementation of REACT. The compounded images were recorded using 0° (Fig. 3b), 2.5° (Fig. 3c), 5° (Fig. 3d), 15° (Fig. 3e), and 30° (Fig. 3f) elevational angular deflections. Compounding at 0° (Fig. 3b) results in minimal image improvement over the uncompounded image (Fig. 3a). Increasing the elevational compounding angle results in a decrease in speckle noise, but also a loss of image fidelity at high angles (15° and 30°) due to increasing elevational angular width and a concomitant increase in interference from structures in adjacent elevational planes (Fig. 3e,f), which manifest as a loss of definition of the water bead boundaries (blue arrow). Visual inspection of the ultrasound images in Fig. 3a–f reveals that 5° EAC yields the optimum balance between speckle noise reduction and image fidelity. The edges of the holes and fine structures around them are preserved and are clearest in 5° EAC (highlighted by the red arrow in Fig. 3d).

To quantify the effect of the elevational angular deflection on the speckle reduction efficiency and image fidelity in REACT, SNR was calculated using a region within the solid phantom (Fig. 3a, red dashed circle) and CNR was calculated using regions within and outside the solid region (Fig. 3a, white and red dashed circles); SNR and CNR were then plotted as a function of the number of compounded images (Fig. 3g,h). As expected, SNR increases with the number of compounded images for all cases, with the rate of SNR improvement increasing with the elevation deflection angle. At the upper end, the SNR for 30° EAC is 3.1 times greater than 0° EAC, after 100 images were compounded (SNR_0°EAC_ = 4.33, SNR _30°EAC_ = 13.33). This improvement results from the higher variation in the speckle pattern of the captured images within the higher elevational angular deflections. In contrast, CNR increases with an increasing number of compounded images only for the 0°, 2.5°, and 5° cases. CNR predominantly decreases with compounding for the 15° and 30° cases. Therefore, while higher elevational angular deflection results in a decrease in speckle noise (higher SNR), too great an angular deflection can cause severe degradation of image fidelity (lower CNR).

## Discussion

The accurate diagnosis and therapeutic utility of EUS for gastrointestinal disorders is limited by the presence of speckle noise^8,9^, which hinders the identification of gastrointestinal tract layer boundaries with low acoustic impedance differences^8^. To minimize speckle artefacts, compounding methods are preferable to image processing techniques because they yield both high quality images and reveal fine structures obscured by speckle noise, which are otherwise irretrievable using the aforementioned techniques^15,16^. EAC is a preferred spatial compounding technique with both high despeckling efficiency and good temporal resolution, which makes it favorable for real time imaging^18^. However, tight anatomical constraints of hollow organs prevent implementation of EAC in EUS using 2D or tilting 1D transducer arrays. Here, we demonstrated a novel deployment of spatial compounding in radial EUS by implementing REACT in a radial geometry, which can lead to image quality improvements in clinical EUS and enable more accurate diagnoses of GI lesions.

Visual and quantitative inspection of the ultrasound images from a cylindrical layered phantom (Fig. 2) illustrates that spatial compounding in radial EUS can provide more efficient speckle reduction with retrieved fine structures, which are not recoverable with commonly used image post-processing techniques such as Frost filtering. As expected, the wider the elevational angle employed, the greater the speckle suppression in the compounded image, which translates to higher SNRs (Fig. 3g). Compounded images acquired within elevational angular deflections of 0°, 2.5°, 5°, 15°, and 30° yielded SNR improvements of 1.27, 2.19, 2.68, 3.32, and 3.91-fold, respectively, compared to the single image (SNR_single_ = 3.41, SNR_0° EAC_ = 4.33, SNR_2.5° EAC_ = 7.47, SNR _5° EAC_ = 9.14, SNR _15° EAC_ = 11.32, SNR _30° EAC_ = 13.33). The SNR enhancement is in agreement with previously reported findings using a linear configuration of REACT^18^. As expected, the similar SNRs obtained for averaging (Fig. 2d) and 0° EAC (Fig. 2e) confirmed that the uncorrelated speckle patterns are produced by effectively changing the elevational angular deflection using an acoustic refractive lens. Moreover, SNR in REACT has an increasing trend by increasing the number of images, yet at a greater rate for the 30° EAC case (Fig. 3g), owing to the widest elevational angular width and therefore providing images with less correlated speckle patterns.

Although higher elevational angular deflection results in higher despeckling efficiency, a trade-off between speckle reduction and image distortion determines the optimal compounding angle for the imaging target of interest^18^. In agreement with our previous study^18^, we found that out-of-plane signals can also degrade the quality of the elevational compounded image for radial EUS. This is relevant in particular when imaging small organs such as lymph nodes adjacent to the GI tract. We demonstrate an increase in SNR with wider elevational angular deflection in a phantom containing lymph node like structures; however, angular deflection above 5° results in overall degradation of CNR (Fig. 3). This drop in CNR is due to the out-of-plane artefacts and represents a loss of image contrast and resolution, which can affect accurate diagnosis. Our experiments suggest that 5° EAC provides an optimal trade-off for high despeckling efficiency with minimal image fidelity loss for lymph node-like structures (Fig. 3).

This study demonstrates REACT as a first potential compact and low-cost solution to impart current radial echo-endoscopes with spatial compounding. The optimum elevational angular deflection for imaging lymph nodes was also investigated to achieve the best combination of despeckling efficiency and high image fidelity, required for accurate identification of pathological lymph nodes in the GI tract. However, the optimum extended elevational angular deflection that can provide high despeckling efficiency while preserving image fidelity in EUS depends on the size and depth of the organ imaged within the body. Hence, further studies are required to define the best organ-specific angular deflections. The lens material has an effect on SNR as can be seen by individual images captured with (Fig. 2c) and without (Fig. 2b) the acoustic lens. This is caused by the acoustic attenuation and reflection and could be diminished by either utilizing lower attenuating materials to manufacture the lens compared to PMMA (e.g. TPX) or diffractive lenses with lower effective thicknesses. To enable real-time despeckling in radial EUS using REACT, the translation of the lens should be automated to allow frame rates as high as the acquisition speed of the US imaging system. Future work will aim to translate REACT into clinical settings by miniaturizing and integrating the acoustic lens to the existing radial echo-endoscopes.

In summary, we demonstrate that REACT is ideally suited for radial EUS and can uniquely impart spatial compounding to radial ultrasound endoscopy for the first time, enabling observation of fine structures hidden by speckle noise. This low-cost and simple spatial compounding method can be of great benefit in clinics to improve image quality and contrast in current radial echo-endoscopes to heighten the accuracy in visualization and identification of pathological lymph nodes in staging of gastrointestinal tract cancers.

## Supplementary Information

Supplementary Information.

## Data Availability

The datasets generated during and/or analysed during the current study are available from the corresponding author on reasonable request.
