# Supplementary material for: Speckle reduction in ultrasound endoscopy using refraction based elevational angular compounding
Source: Sci Rep. 2021 Sep 15;11:18370. doi: 10.1038/s41598-021-97717-2 (PMC8443636; doi:10.1038/s41598-021-97717-2)
Supplement: Supplementary file 1 — Supplementary Information. [file 41598_2021_97717_MOESM1_ESM.docx]

**SUPPLEMETARY INFORMATION**

To validate our theoretical design calculations for the desired refraction of each manufactured lens (Snell’s law), we characterized the elevational angular view of the 30˚ EAC lens using a metal target (see our previous work^18^) and a needle hydrophone sensor for confirmation. A metal target (hex key, ~2 mm diameter) was attached to a XYZ micrometer stage, positioned vertically in front of the transducer, and monitored in real time on the US image. The target was moved to a position towards the edge of the ring transducer, such that it was barely visible in the image. The relative distance between the transducer and the target was measured and the inherent elevational angle of the transducer calculated using simple trigonometry, yielding θ_i_ = 29.4˚. The manufactured acoustic lens was subsequently inserted between the transducer and the target. The lens was translated until the target reappeared in the image due to acoustic refraction. The target was then moved vertically outwards until it just disappeared from the image. This process was iterated until the target no longer became visible after repositioning the acoustic refraction lens. The new relative distance between the transducer and the target were measured and utilized to calculate the extended elevational angle of θ_E_ = 59.1˚ and subsequently the lens's effective maximum elevational angular deflection of θ_E_ – θ_i_ =29.7˚ (Fig. 1a-c in supplementary). A similar iterative measurement process was performed to find the outer most position where a needle hydrophone could detect, through the 30° EAC lens, the pulses emitted by the transducer. This point corresponds to the maximum possible deflection of the lens, and the subtended angle was calculated by simple trigonometry using the relative distance between the needle and the edge of the transducer, yielding, for this lens, an extended elevational angle θ_E_ = 59.3˚ (Fig. 1d in supplementary). The subtraction of the inherent and extended elevational angle yielded to the effective maximum elevational angular deflection of 29.9˚. Additionally, we recorded the maximum voltage sensed by the needle hydrophone as the lens was translated through its full length to confirm the refractive capability of the lens (Fig. 1e in supplementary).


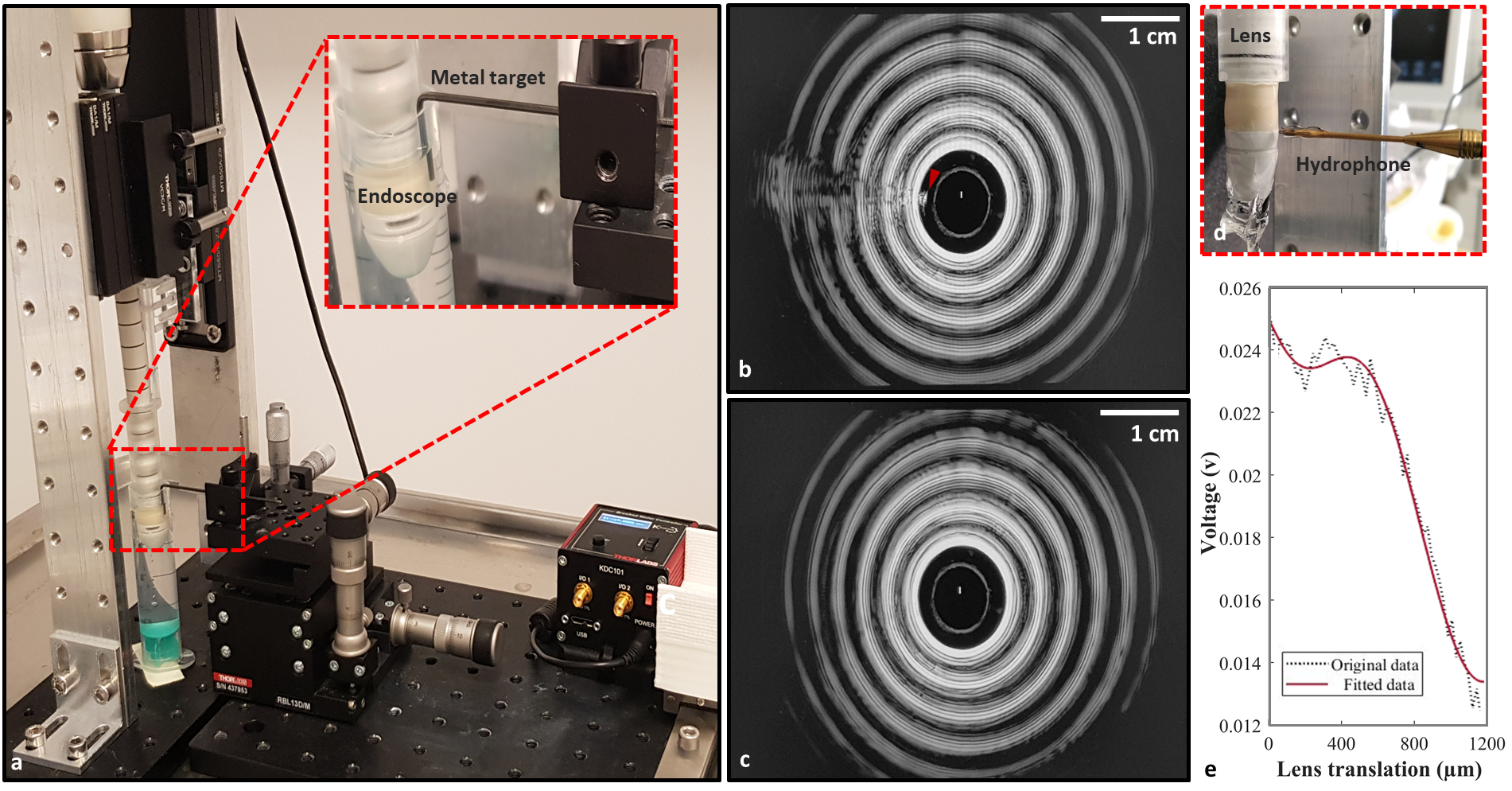


**Supplementary Fig.1** Elevational angular characterization of the manufactured lenses based on hydrophone measurements and the proposed sliding metal target method. **(a)** Photograph of the characterization set-up used for both approaches. The picture inset shows a detailed view of the sliding metal target in front of the top edge of the radial endoscope transducer, inside the water tank. **(b), (c)** Ultrasound images showing the absence and presence of the metal target in the transducer field of view, respectively. The red arrow points to the tip of metal target. **(d)** Detailed picture of the hydrophone placed on the lower edge of the radial endoscope transducer, coupled with ultrasonic gel. **(e)** Hydrophone maximum voltage at each position of the translating 30° EAC lens.

Supplementary Fig. [2](https://www.nature.com/articles/s41598-020-75092-8#Fig4)a,b display the variation in pixel intensity for images compounded using 0° and 5° EAC lens, which was mapped to explore the speckle pattern decorrelation between individual recorded images (Fig. [2](https://www.nature.com/articles/s41598-020-75092-8#Fig4)e,g). As predicted, images taken inside the 5° elevational angular deflection have stronger speckle pattern decorrelation, resulting in greater speckle reduction, as seen in Fig. 2g. To investigate the effect of step size on image decorrelation of the speckle pattern when translating the 5˚ and 0˚ EAC lens, we performed a correlation coefficient analysis of the captured images. Decorrelation from consecutive image pairs (e.g., 1 vs. 2, 2 vs. 3, etc.; supplementary Fig. 2c) was used to determine which region of the lens afforded the most effective decorrelation. A correlation coefficient calculation was also performed between an arbitrary image (here, image number 2) and each of the 100 acquired images to confirm that the center of the lens was in the optimum position to capture images with maximum decorrelation (e.g., 2 vs. 1, 2 vs. 2, 2 vs. 3, etc.; supplementary Fig. 2d, respectively). We observed that a similar despeckling power could be achieved by compounding fewer images at unequal step sizes compared to 100 images with equal step sizes through linear translation of the lens. The optimized step size would be achieved by linearly translating the lens to positions that provide equidistant acoustic beam deflections.


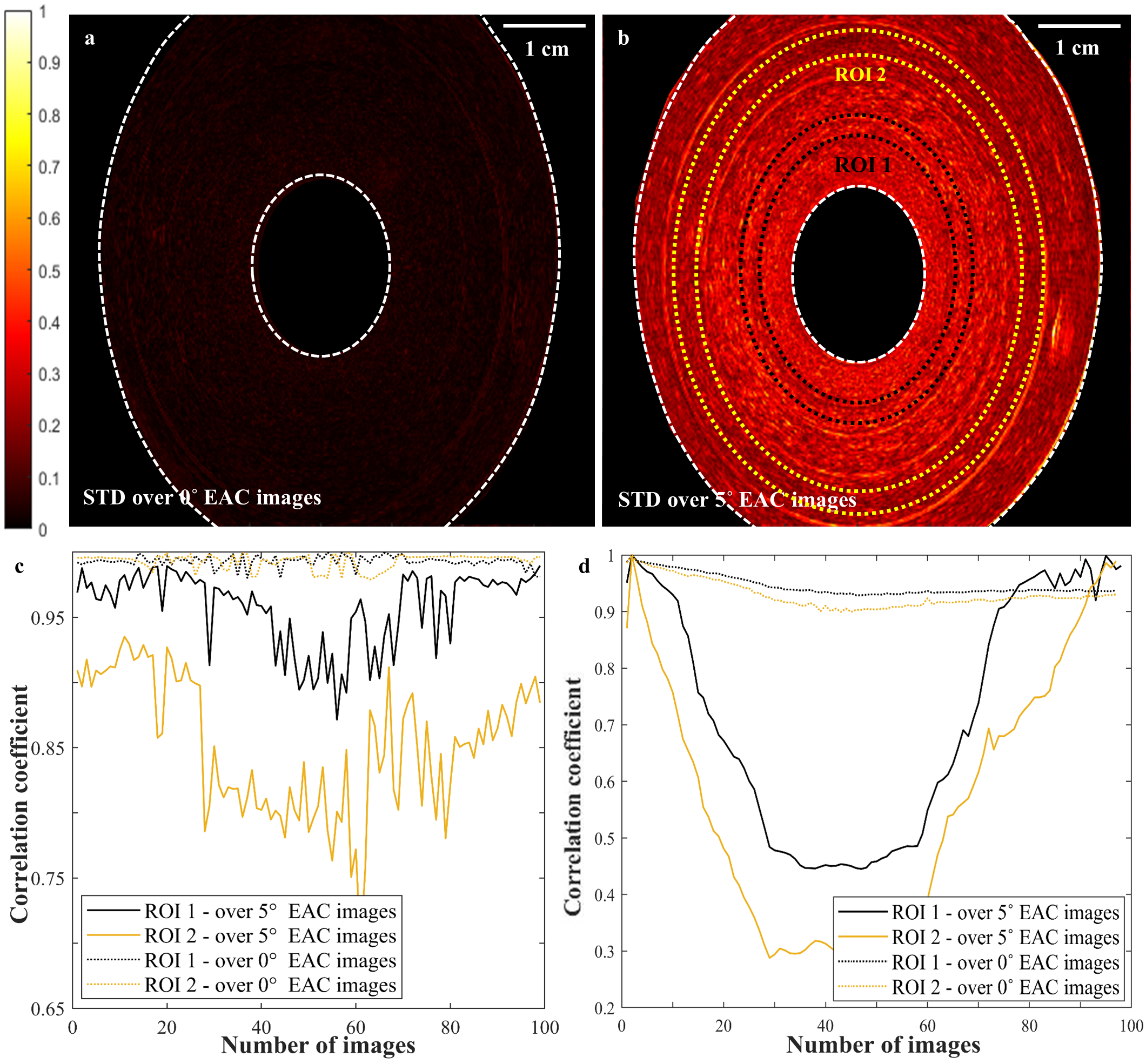


**Supplementary Fig. 2** Elevational angular deflection results in speckle pattern decorrelation. Intensity of STD maps at pixel-level along all captured images with **(a)** 0° and **(b)** 5° EAC lenses, respectively; white dashed lines delineate the phantom inner and outer surface contours. Regions enclosed by the black (ROI 1) and yellow (ROI 2) circular rings used to derive the correlation coefficient shown in panel **c** and **d**. Correlation coefficient between **(c)** consecutive image pairs (e.g., 1 vs 2, 2 vs 3, etc.) and **(d)** an arbitrary selected image (here, image number 2) and each of the 100 captured images (e.g. 2 vs 1, 2 vs 2, 2 vs 3, etc.), resulting from the translation of the 5° and 0° EAC lens at equal step sizes in front of the transducer.
